# Extrapolating echocardiographic determinants of elevated Left Atrial Pressure (LAP) to Cardiac Magnetic Resonance Imaging (CMR) to determine the best CMR correlate of elevated LAP

**DOI:** 10.1186/1532-429X-18-S1-P248

**Published:** 2016-01-27

**Authors:** Mohan Mallikarjuna Rao Edupuganti, Srikanth Vallurupalli, Sabha Bhatti, Shelly Lensing, Tarun Pandey

**Affiliations:** 1 grid.241054.60000000122929177Radiology, University of Arkansas for Medical Sciences, Little Rock, AR USA; 2 grid.241054.60000000122929177Cardiology, University of Arkansas for Medical Sciences, Little Rock, AR USA; 3 grid.241054.60000000122929177Biostatistics, University of Arkansas for Medical Sciences, Little Rock, AR USA

## Background

The ratio of the transmitral early inflow wave velocity (E wave velocity) and the velocity of the septal (or lateral) mitral annulus as measured by tissue Doppler (the e' wave velocity) can be used to estimate left atrial pressure on echocardiographic examination. An elevated E/e' (more than 15 when using the septal mitral annular velocity) is an established measure of elevated LAP.

Furthermore when the left atrial pressure rises, simultaneous changes take place in the pulmonary venous flow. The normal flow pattern in the pulmonary veins consist of a prominent systolic and a smaller diastolic component with the ratio of the peak systolic to diastolic velocities more than 1. With elevated left atrial pressure the ratio drops to below 1.

## Hypothesis

We hypothesize that echocardiographic techniques of measuring LAP can be extrapolated to cardiac CMR and should correlate well with echo derived E/e'.

## Methods

47 (23 female, 24 male) patient had simultaneous assessment of cardiac function with CMR and echocardiograms. The average age of the group was 59 years. The degree of correlation for the following were measured using Spearman's rank correlation:

- CMR derived S/D ratio with echo derived E/e' and
- CMR derived E/e' with echo derived E/e' .

We then divided the patients into a low LAP and a high LAP group based on echocardiographic E/e' less than and more than 15 respectively. 32 patients were in the low LAP group and 15 were in the high LAP group. We repeated the above analysis for each group to determine if the degree of correlation between CMR derived S/D ratios and E/e' values and echo derived E/e' values persisted across all values of LAP.

## Results

Overall, Spearman's rank correlation was 0.65 for Echo vs. CMR E/e' (n = 31, p < 0.001) and -0.39 for Echo E/e' vs. CMR S/D (n = 30, p = 0.032). At low LAP, the correlation was 0.30 for Echo vs. CMR E/e' (n = 18, P=0.233) and 0.28 for ECHO E/e' vs. CMR S/D (n = 17, p = 0.277). At high LAP, the correlation was 0.08 for Echo vs. CMR E/e' (n = 13, P=0.803) and -0.12 for Echo E/e' vs. CMR S/D ratio (n = 13, p = 0.707).

## Conclusions

When compared to echo, CMR derived E/e' is a better measure of LAP than pulmonary vein S/D ratio. Furthermore the CMR measures of LAP appear to correlate better with echocardiographic determination of LAP at lower LAP values compared to higher LAP values. While overall correlations between CMR and echo parameters were statistically significant, our study could not establish significance between these parameters in smaller groups mainly due to the small numbers in our study population. Our results are encouraging and further studies with a larger sample of patients should be undertaken to define the best CMR correlate of LAP.

